# Supplementary material for: Analysis of transcriptional changes in the immune system associated with pubertal development in a longitudinal cohort of children with asthma
Source: Nat Commun. 2023 Jan 16;14:230. doi: 10.1038/s41467-022-35742-z (PMC9842661; doi:10.1038/s41467-022-35742-z)
Supplement: Supplementary file 1 — Supplementary information [file 41467_2022_35742_MOESM1_ESM.pdf]

Supplementary Figures

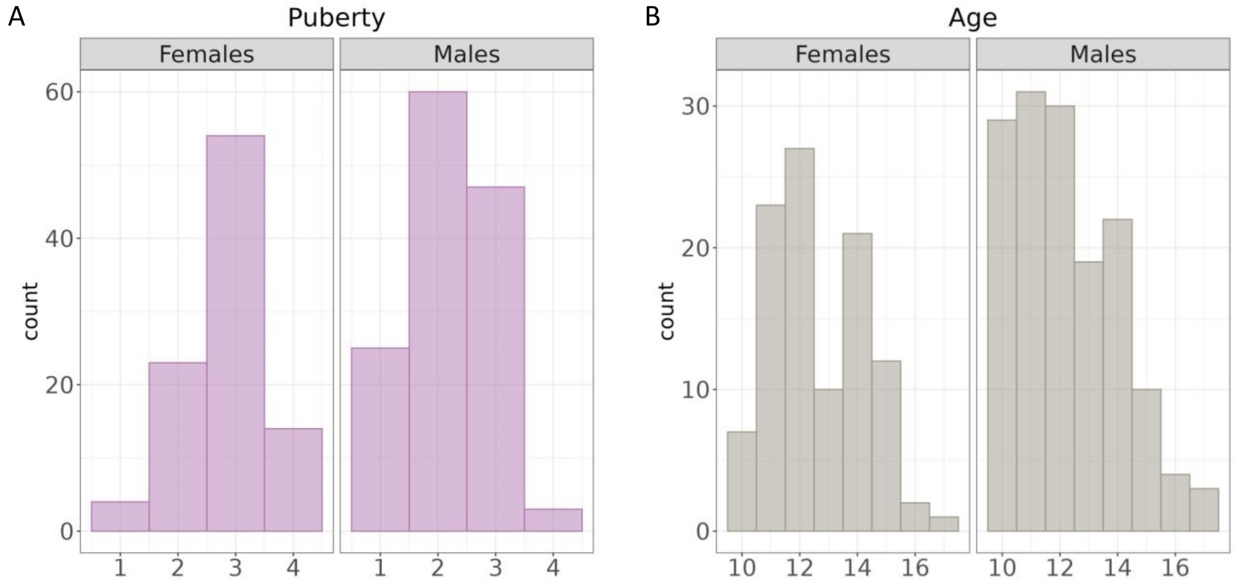

Fig. S1. Distribution of puberty and age in the cross-sectional sample. A – Histogram representing the distribution of pubertal development stages in females (left panel) and males (right panel), B - Histogram representing the distribution of age in years in females (left panel) and males (right panel).

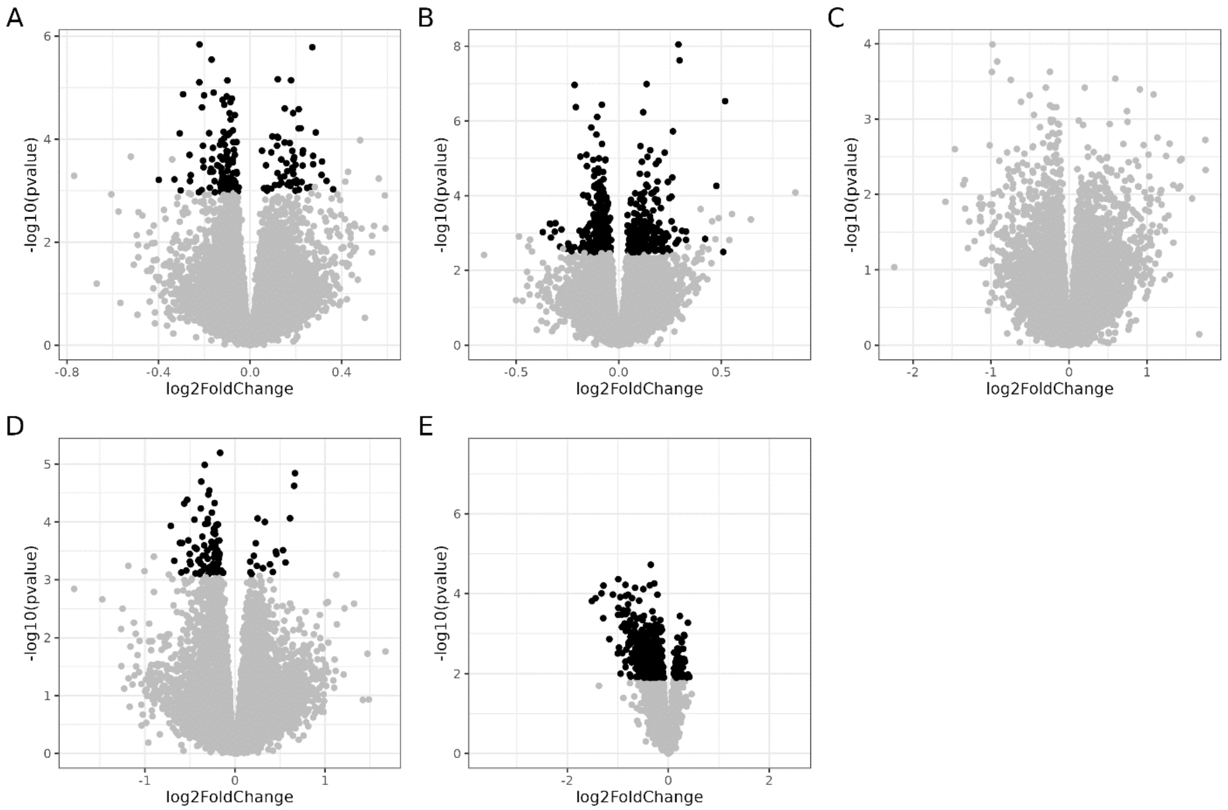

Fig. S2. Volcano plots of differential gene expression (DGE) analysis effect size (log2 fold-change, x axis) and -log10 of p-values (y axis). A – longitudinal DGE analysis across time in females, B – longitudinal DGE analysis across time in males, C – longitudinal DGE analysis across puberty stages in females, D – longitudinal DGE analysis across puberty stages in males, E – cross-sectional DGE analysis of pre- and post-menarche in females. Black denotes significant values (10% FDR), grey denotes not significant, according to Wald test with Benjamini-Hochberg multiple test correction.

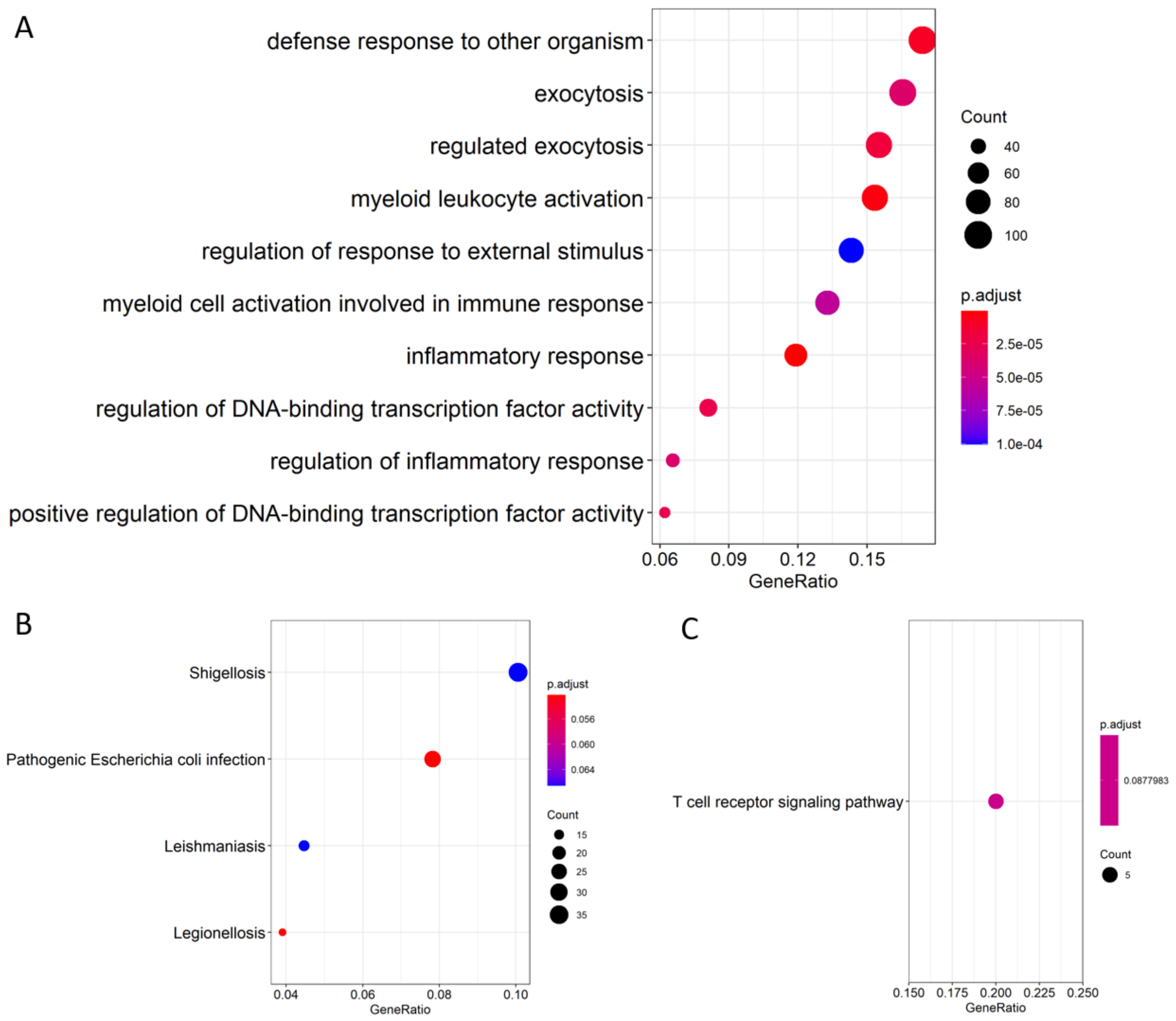

Fig. S3. Biological processes and pathway enriched within genes whose expression differs between pre- and post-pubertal females. A - dotplot represents enrichment of Gene Ontology biological processes within genes whose expression is lower in females post menarche, B – dotplot represents enrichment of KEGG pathways within genes whose expression is lower in females post menarche, C – dotplot represents enrichment of KEGG pathways within genes whose expression is higher in females post menarche. Over-representation analysis performed using a hypergeometric test with Benjamini-Hochberg adjustment for multiple comparisons.

Supplementary Tables

Table S1. Sample size. Reported are the sample size (N) and age range for each analysis, and age range for first timepoint (T0) and second timepoint (T1) of the longitudinal analysis).

**Age**					
**Subset**	**N (Cross-Sectional)**	**Age Range**	**N (Longitudinal)**	**Age Range T0**	**Age Range T1**
Total	251	10-17 yrs	163	10-15 yrs	11-16 yrs
Males	148	10-17 yrs	97	10-15 yrs	11-16 yrs
Females	103	10-17 yrs	66	10-15 yrs	11-16 yrs
**Puberty**					
**Subset**	**N (Cross-Sectional)**	**Age Range**	**N (Longitudinal)**	**Age Range T0**	**Age Range T1**
Total	240	10-17 yrs	142	10-15 yrs	11-16 yrs
Males	141	10-17 yrs	85	10-15 yrs	11-16 yrs
Females	99	10-17 yrs	57	10-15 yrs	11-16 yrs
**Menarche**					
**Subset**	**N (Cross-Sectional)**	**Age Range**			
Females	66	10-16 yrs			

Table S2. Pubertal Development Questionnaire.

Question	Answers	Numerical value
**Females' questionnaire**		
Would you say your growth in height (getting taller)…	Has not yet began to spurt ('spurt' means more growth than usual)	1
	Has barely started to spurt	2
	Has definitely started to spurt, but has not finished	3
	Seems complete (you're about as tall as you're going to get)	4
How about the growth of your body hair? ("Body hair" means hair any place other than your head, such as under your arms).	Has not started growing	1
	Has barely started growing	2
	Has definitely started growing, but has not finished	3
	Seems complete (you have as much body hair as you're going to get)	4
Have you noticed any skin changes, especially pimples?	Skin has not yet started showing changes	1
	Skin has barely started showing changes	2
	Skin changes have definitely started but are not finished	3
	Skin changes seem complete	4
Have you noticed that your breasts have begun to grow?	Have not yet started growing	1
	Have barely started growing	2
	Breast growth has definitely started, but is not finished	3
	Breast growth seems completed	4
Have you begun to menstruate? ("menstruate" means to get your period)	Yes	4
	No	1
**Males' questionnaire**		
Would you say your growth in height (getting taller)…	Has not yet began to spurt ('spurt' means more growth than usual)	1
	Has barely started to spurt	2
	Has definitely started to spurt, but has not finished	3
	Seems complete (you're about as tall as you're going to get)	4
How about the growth of your body hair? ("Body hair" means hair any place other than your head, such as under your arms).	Has not started growing	1
	Has barely started growing	2
	Has definitely started growing, but has not finished	3
	Seems complete (you have as much body hair as you're going to get)	4
Have you noticed any skin changes, especially pimples?	Skin has not yet started showing changes	1
	Skin has barely started showing changes	2
	Skin changes have definitely started but are not finished	3
	Skin changes seem complete	4
Have you noticed a deepening of your voice?	Voice has not yet started changing	1
	Voice has barely started changing	2

	Voice has definitely started changing, but is not finished	3
	Voice change seems complete	4
Have you started to grow hair on your face?	Facial hair has not started growing	1
	Facial hair has barely started growing	2
	Facial hair growth has definitely started but is not finished	3
	Facial hair growth seems complete	4

Supplementary Files

Supplementary Data 1. Results of longitudinal differential gene expression analysis across time in females. Analysis performed in DESeq2 as described in Methods using Wald test with Benjamini-Hochberg multiple test correction.
http://genome.grid.wayne.edu/puberty/S1_cage1_Female_stats_longit.xlsx

Supplementary Data 2. Results of longitudinal differential gene expression analysis across time in males. Analysis performed in DESeq2 as described in Methods using Wald test with Benjamini-Hochberg multiple test correction.
http://genome.grid.wayne.edu/puberty/S2_cage1_Male_stats_longit.xlsx

Supplementary Data 3. Results of cross-sectional differential gene expression analysis across age in females. Analysis performed in DESeq2 as described in Methods using Wald test with Benjamini-Hochberg multiple test correction.
http://genome.grid.wayne.edu/puberty/S3_cage1_Female_stats_cs.xlsx

Supplementary Data 4. Results of cross-sectional differential gene expression analysis across age in males. Analysis performed in DESeq2 as described in Methods using Wald test with Benjamini-Hochberg multiple test correction.
http://genome.grid.wayne.edu/puberty/S4_cage1_Male_stats_cs.xlsx

Supplementary Data 5. Significance of longitudinal multivariate adaptive shrinkage analysis across time in both sexes (LFSR).
http://genome.grid.wayne.edu/puberty/S5_cage1_LFSR.xlsx

Supplementary Data 6. Effect size estimates from longitudinal multivariate adaptive shrinkage analysis across time in both sexes.
http://genome.grid.wayne.edu/puberty/S6_cage1_beta.xlsx

Supplementary Data 7. Results of longitudinal differential gene expression analysis across puberty stages in females. Analysis performed in DESeq2 as described in Methods using Wald test with Benjamini-Hochberg multiple test correction.
http://genome.grid.wayne.edu/puberty/S7_cgpd_stats_longit.xlsx

Supplementary Data 8. Results of longitudinal differential gene expression analysis across puberty stages in males. Analysis performed in DESeq2 as described in Methods using Wald test with Benjamini-Hochberg multiple test correction.
http://genome.grid.wayne.edu/puberty/S8_cbpd_stats_longit.xlsx

Supplementary Data 9. Results of cross-sectional differential gene expression analysis across puberty stages in females. Analysis performed in DESeq2 as described in Methods using Wald test with Benjamini-Hochberg multiple test correction.
http://genome.grid.wayne.edu/puberty/S9_cgpd_stats_cs.xlsx

Supplementary Data 10. Results of cross-sectional differential gene expression analysis across puberty stages in males. Analysis performed in DESeq2 as described in Methods using Wald test with Benjamini-Hochberg multiple test correction.

http://genome.grid.wayne.edu/puberty/S10_cbpd_stats_cs.xlsx

Supplementary Data 11. Results of cross-sectional differential gene expression of pre and post-menarche in females. Analysis performed in DESeq2 as described in Methods using Wald test with Benjamini-Hochberg multiple test correction.
http://genome.grid.wayne.edu/puberty/S11_cgpd5_stats_cs.xlsx

Supplementary Data 12. Results of cis interaction eQTL mapping. Coefficients are derived from linear regression as described in Methods; p-values are derived from two-sided t-test.
http://genome.grid.wayne.edu/puberty/S12_GxPuberty_all.xlsx

Supplementary Data 13. Results of Transcriptome-Wide Association Study of age at menarche. Z test was used for significance testing followed by Bonferroni multiple testing correction.
http://genome.grid.wayne.edu/puberty/S13_AAM-TWAS_Blood.xlsx

Supplementary Data 14. Overlap of genes associated with age at menarche via TWAS and differentially expressed genes.
http://genome.grid.wayne.edu/puberty/S14_AAM-TWAS_DEG_overlap.xlsx

Supplementary Data 15. Overlap of genes associated with asthma via TWAS (Zhang et al, 2019) and differentially expressed genes.
http://genome.grid.wayne.edu/puberty/S15_Astma-TWAS_DEG_overlap.xlsx
