# Supplementary material for: Analysis of transcriptional changes in the immune system associated with pubertal development in a longitudinal cohort of children with asthma
Source: Nat Commun. 2023 Jan 16;14:230. doi: 10.1038/s41467-022-35742-z (PMC9842661; doi:10.1038/s41467-022-35742-z)
Supplement: Supplementary file 3 — Description of Additional Supplementary Files [file 41467_2022_35742_MOESM3_ESM.pdf]

Description of Additional Supplementary Files

File Name: Supplementary Data 1
Description: Results of longitudinal differential gene expression analysis across time in females.

File Name: Supplementary Data 2
Description: Results of longitudinal differential gene expression analysis across time in males.

File Name: Supplementary Data 3
Description: Results of cross-sectional differential gene expression analysis across age in females.

File Name: Supplementary Data 4
Description: Results of cross-sectional differential gene expression analysis across age in males.

File Name: Supplementary Data 5
Description: Significance of longitudinal multivariate adaptive shrinkage analysis across time in both sexes (LFSR).

File Name: Supplementary Data 6
Description: Effect size estimates from longitudinal multivariate adaptive shrinkage analysis across time in both sexes.

File Name: Supplementary Data 7
Description: Results of longitudinal differential gene expression analysis across puberty stages in females.

File Name: Supplementary Data 8
Description: Results of longitudinal differential gene expression analysis across puberty stages in males.

File Name: Supplementary Data 9
Description: Results of cross-sectional differential gene expression analysis across puberty stages in females.

File Name: Supplementary Data 10
Description: Results of cross-sectional differential gene expression analysis across puberty stages in males.

File Name: Supplementary Data 11
Description: Results of cross-sectional differential gene expression of pre and post-menarche in females.

File Name: Supplementary Data 12
Description: Results of cis interaction eQTL mapping.

File Name: Supplementary Data 13
Description: Results of Transcriptome-Wide Association Study of age at menarche.

File Name: Supplementary Data 14
Description: Overlap of genes associated with age at menarche via TWAS and differentially expressed genes.

File Name: Supplementary Data 15
Description: Overlap of genes associated with asthma via TWAS (Zhang et al, 2019) and differentially expressed genes.
